# Activation of cyclin-dependent kinase 5 mediates orofacial mechanical hyperalgesia

**DOI:** 10.1186/1744-8069-9-66

**Published:** 2013-12-21

**Authors:** Michaela Prochazkova, Anita Terse, Niranjana D Amin, Bradford Hall, Elias Utreras, Harish C Pant, Ashok B Kulkarni

**Affiliations:** 1 Functional Genomics Section, Laboratory of Cell and Developmental Biology, National Institute of Dental and Craniofacial Research, National Institutes of Health, Bethesda, MD 20892, USA; 2 Laboratory of Neurochemistry, National Institute of Neurological Disorders and Stroke, National Institutes of Health, Bethesda, MD 20892, USA; 3 Laboratory of Cellular and Neuronal Dynamics, Faculty of Science, University of Chile, Santiago, Chile

**Keywords:** Cdk5, p35, Trigeminal ganglia, Orofacial pain, Mouse model

## Abstract

**Background:**

Cyclin-dependent kinase 5 (Cdk5) is a unique member of the serine/threonine kinase family. This kinase plays an important role in neuronal development, and deregulation of its activity leads to neurodegenerative disorders. Cdk5 also serves an important function in the regulation of nociceptive signaling. Our previous studies revealed that the expression of Cdk5 and its activator, p35, is upregulated in nociceptive neurons during peripheral inflammation. The aim of the present study was to characterize the involvement of Cdk5 in orofacial pain. Since mechanical hyperalgesia is the distinctive sign of many orofacial pain conditions, we adapted an existing orofacial stimulation test to assess the behavioral responses to mechanical stimulation in the trigeminal region of the transgenic mice with either reduced or increased Cdk5 activity.

**Results:**

Mice overexpressing or lacking p35, an activator of Cdk5, showed altered phenotype in response to noxious mechanical stimulation in the trigeminal area. Mice with increased Cdk5 activity displayed aversive behavior to mechanical stimulation as indicated by a significant decrease in reward licking events and licking time. The number of reward licking/facial contact events was significantly decreased in these mice as the mechanical intensity increased. By contrast, mice deficient in Cdk5 activity displayed mechanical hypoalgesia.

**Conclusions:**

Collectively, our findings demonstrate for the first time the important role of Cdk5 in orofacial mechanical nociception. Modulation of Cdk5 activity in primary sensory neurons makes it an attractive potential target for the development of novel analgesics that could be used to treat multiple orofacial pain conditions.

## Background

Orofacial pain affects millions of people worldwide. It is characterized by throbbing, sharp or burning pain in the head, neck, face, mouth, gums or teeth. Epidemiological studies indicate that orofacial pain occurs in approximately 10% of the adult population [1], and women are more often affected than men by a ratio of 2:1 [2]. Orofacial pain episodes are usually very debilitating for the patient. However, relatively few studies are focused on characterizing orofacial pain, particularly due to the limited number of animal models available to study nociception in the trigeminal region. Most of these models have been adapted from those used for studying peripheral pain and are primarily based on the induction of inflammation by the administration of nociceptive agents, such as complete Freund’s adjuvant [3,4], carrageenan [5,6], and formalin [7-10]. Other models are based on the direct damage to a nerve (cutting, ligating, or crushing) [11-14]. These models suffer from certain limitations, such as variation in subjective observation, inability to escape from the noxious stimulus, and induction of the stress in a test animal. The recently reported operant behavioral assay using a reward-conflict paradigm wherein a test animal can decide between receiving a reward or escaping an aversive stimulus present new perspectives on measuring pain in the orofacial region [15-18].

There is accumulating evidence that protein kinases are involved in mediating several types of pain. Cdk5 is a serine/threonine kinase widely distributed in different mammalian tissues, but its kinase activity is observed primarily in neuronal cells, due to the selective expression of its activators, p35 and p39. Cdk5 plays important roles in many vital processes, including brain development and function, neuronal migration, neurotransmitter release, cell adhesion and survival, drug addiction, learning, memory [19-23], and also in many non-neuronal functions [24-26]. Cdk5 knockout mice are embryonic lethal with numerous lesions in the central nervous system [27]. Efforts to delineate molecular roles of Cdk5 *in vivo* led to the generation of mice overexpressing [28] or lacking [29] p35, an activator of Cdk5. Recently, we and others discovered that Cdk5 activity regulates peripheral pain signaling, and that it is required for the basal responses to noxious heat [30,31]. The p35 knockout mice (with residual Cdk5 activity) showed delayed responses to painful thermal stimulation (hypoalgesia), whereas mice overexpressing p35 (with significantly increased Cdk5 activity) were more sensitive to painful thermal stimulation showing hyperalgesia. Moreover, we have identified that the expression of p35, as well as Cdk5 kinase activity, is present in the dorsal root ganglia and trigeminal ganglia neurons, and both are significantly increased upon the induction of peripheral inflammation [32]. Furthermore, nociceptor-specific Cdk5 conditional knockout mice developed hypoalgesia associated with reduced phosphorylation of the TRPV1 channel [30].

The goal of the current study was to evaluate the role of Cdk5 in orofacial mechanosensation and to characterize the behavioral changes of mice lacking or overexpressing p35 using adapted orofacial stimulation test (Additional file 1).

## Results

### Cdk5 activity in transgenic p35 (Tgp35) and p35 knockout (p35−/−) mice

**Figure 1:**
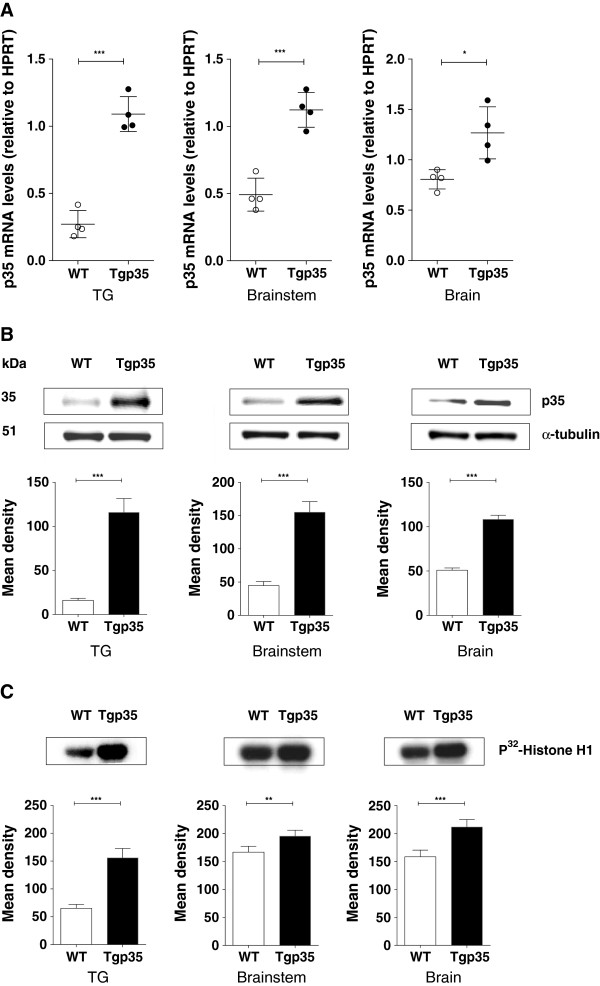
**Analysis of the p35 expression profile in transgenic p35 (Tgp35) mice.** The p35 expression levels and Cdk5 activity in the trigeminal ganglia, brainstem, and brain of the transgenic p35 mice: **(A)** q-PCR analysis revealed significantly enhanced levels of p35 mRNA in the trigeminal ganglia, brainstem, and the brain of the Tgp35 mice. Each data set was normalized to the expression seen in control wild-type animals. The results obtained from four different animals are expressed as mean ± SEM and analyzed by an unpaired *t*-test (***p < 0.001). **(B)** Representative Western blots showing p35 protein levels from Tgp35 tissue lysates together with corresponding densitometric analysis. Data were normalized to the levels of p35 in wild-type controls, and are presented as mean ± SEM (unpaired *t*-test, ***p < 0.001). **(C)** Cdk5 activity in the trigeminal ganglia, brainstem, and the brain of the Tgp35 mice. The data are presented as mean ± SEM and analyzed by an unpaired *t*-test (***p < 0.001).

**Figure 2:**
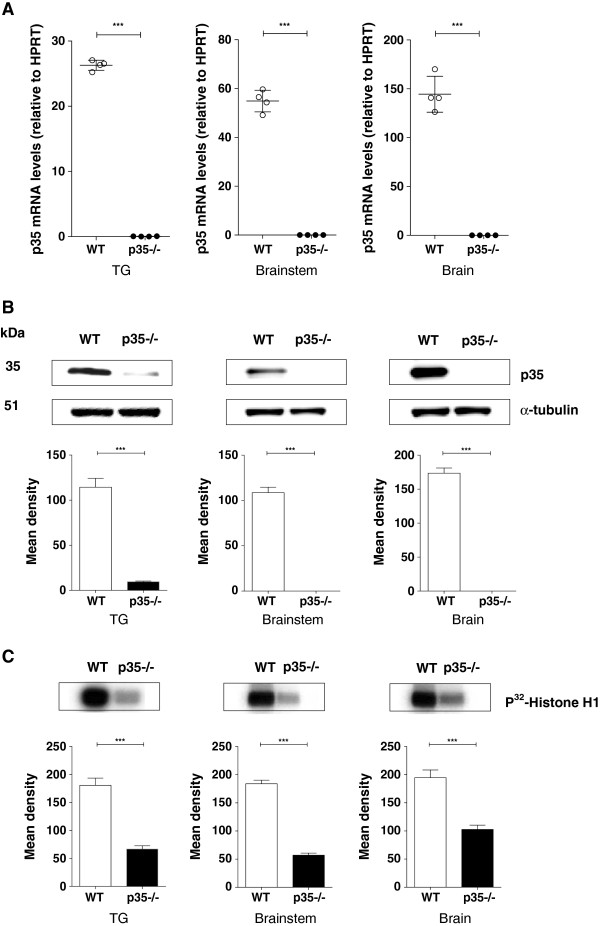
**Analysis of the p35 expression profile in p35 knockout mice.** The p35 expression levels and Cdk5 activity in the trigeminal ganglia, brainstem, and brain of the p35 knockout mice: **(A)** q-PCR analysis revealed significantly decreased levels of p35 mRNA in the trigeminal ganglia, brainstem, and brain of the p35 knockout mice. Each data set was normalized to the expression seen in control wild-type animals. Results obtained from four different animals are expressed as mean ± SEM and analyzed by an unpaired *t*-test (***p < 0.001). **(B)** Representative Western blots showing p35 protein levels from p35 knockout mice together with corresponding, densitometric analysis. Data were normalized to the levels of p35 in wild-type controls, and are presented as mean ± SEM (unpaired *t*-test, ***< 0.001). **(C)** Cdk5 activity in the trigeminal ganglia, brainstem, and brain of the p35 knockout mice. Data are presented as mean ± SEM and analyzed by an unpaired *t*-test (**p < 0.01, ***p < 0.001).

We initially examined the expression and activity of Cdk5/p35 in the trigeminal ganglia, brainstem, and brain of mice that overexpress or lack p35. Analysis of the Tgp35 mice revealed a significant increase in p35 mRNA (Figure 1A) as well as in p35 protein levels (Figure 1B). There was a three-fold increase in Cdk5 activity (Figure 1C) in the trigeminal ganglia of Tgp35 mice compared with the wild-type (WT) mice (p < 0.001). The Tgp35 mice also showed a significant increase in p35 mRNA and protein levels as well as in Cdk5 activity in brainstem and in brain (Figure 1A-C). The analysis of the p35−/− mice showed almost undetectable p35 mRNA and protein levels (Figure 2A and B) and significantly decreased Cdk5 activity (Figure 2C) in tissue homogenates from the trigeminal ganglia, brainstem, and brain, as compared to controls (p < 0.001). The p35 expression levels and Cdk5 activity correlated with the mouse genotype, thus confirming that the p35 level is the limiting factor for the Cdk5 activity [28].

### Normal motor coordination and locomotion in Tgp35 and p35−/− mice

Having established the p35 expression levels and the Cdk5 activity in Tgp35 and p35−/− mice, we determined whether this difference in expression and activity of Cdk5/p35 could affect the motor coordination and locomotion of these genetically modified animals. We did not observe any motor deficit using the acceleration test with rotarod when the rotation was set to accelerate from 4 to 40 rpm during the defined period of time. The average latency to drop from the cylinder was 269 seconds in Tgp35 and 274 seconds in wild-type FVBN control mice, respectively (Additional file 2A). Similarly, no significant difference in the fall latency was observed in p35−/− mice (average latency 253 seconds in p35−/− mice and 256 seconds in the wild-type C57 controls) (Additional file 2A). There were no significant differences in the fall latency during the testing using the constant rotation speed. The p35−/−, Tgp35, and their respective, wild-type controls showed no motor deficits as evident from the mean time spent on the rotarod (Additional file 2B).

### Normal anxiety level and exploratory behavior in Tgp35 and p35−/− mice

Before performing the orofacial operant assay on these mice, we also assessed anxiety, exploratory activity, and stereotypical behavioral using the open-field test. Neither horizontal (Additional file 2C) or vertical (Additional file 2D) activity was affected in the p35−/− or Tgp35 mice, in comparison to the control mice. Since the middle of a non-familiar arena is anxiogenic for rodents, anxiety was studied by analyzing the time spent and total distance travelled in the middle of the cage. There were no significant differences in the time spent in the center of the cage as well as the center distance travelled by the Tgp35 and their littermate controls (Additional file 3A), and the p35−/− and wild-type C57 mice controls (Additional file 4A). There were also no significant changes in the stereotypical behavioral (Additional file 3B and Additional file 4B) or time spent at the different corners of the activity cage (Additional file 3C and Additional file 4C), indicating that the difference in the p35 levels did not cause any change in anxiety or exploratory behavior of the mice.

### Normal baseline behavior in orofacial operant assay

**Figure 3:**
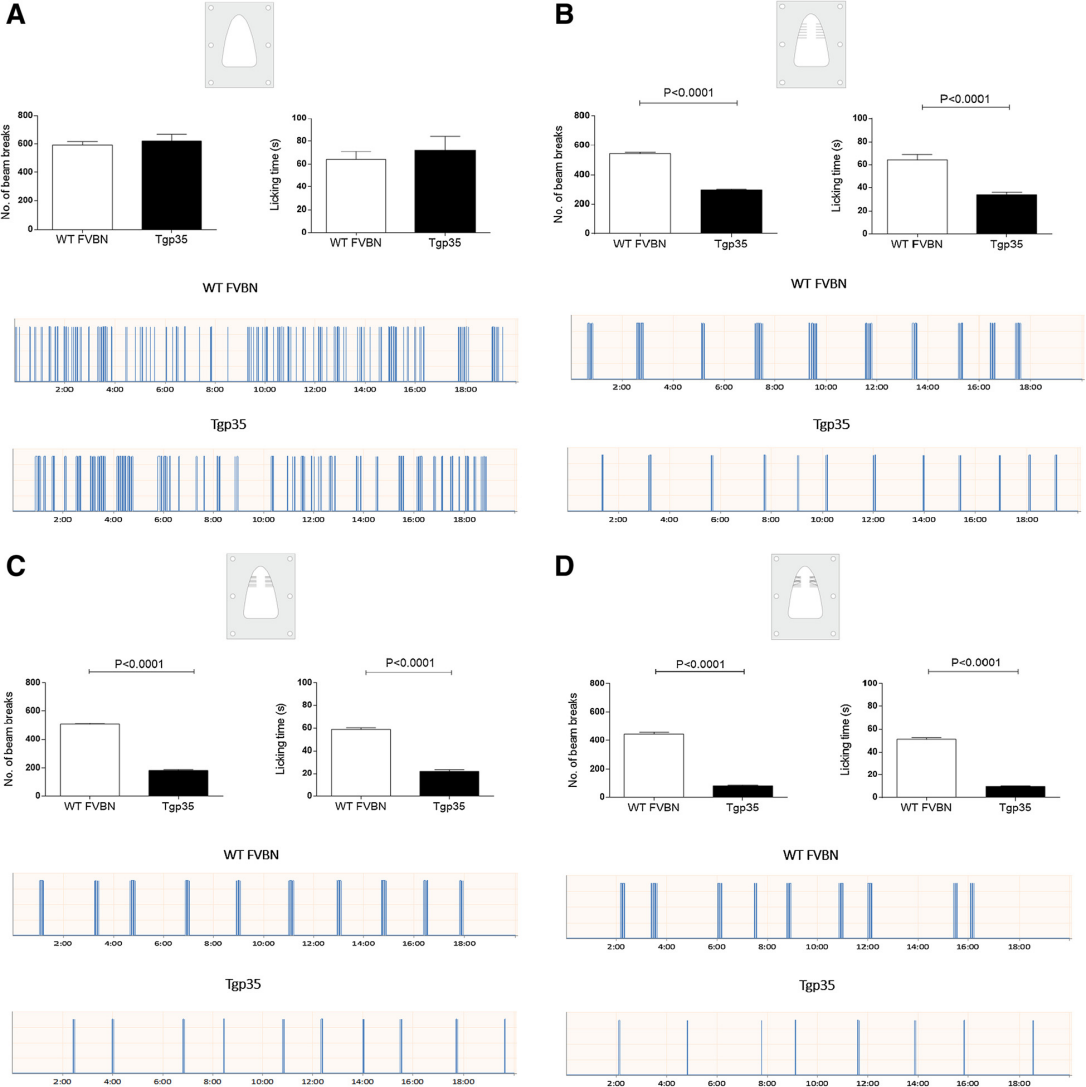
**Licking responses of Tgp35 mice during the orofacial operant assay.** During the testing, the animals accessed the reward by inserting their snout through the plates with different number of wires. This was done in order to apply different force levels to the animal vibrissal pad. The number of beam breaks during the acquisition of the reward together with the licking time was measured: **(A)** without any stimulation, **(B)** using the plate with 6 + 6 wires, **(C)** 9 + 9 wires, and **(D)** 13 + 13 wires. Data are presented as mean ± SEM from four different animals measured five times in case of the baseline and three times after the induction of the trigeminal nociception (unpaired *t*-test, ***p < 0.001). Two sample traces show the automatic recordings of drinking behavior in a testing period of 20 minutes in a single control (upper trace) and single Tgp35 mouse (lower trace).

It was previously reported that the reward conflict paradigm (using parameters such as reward licking events, facial contact events, and facial contact duration) could serve as a characteristic marker of pain in the orofacial area [15,16]. In the mouse orofacial operant assay that we report here, the number of attempts the test mouse made to acquire the reward and the duration that it spent acquiring the reward were the basic outcomes for this behavioral testing. Naïve animals were initially trained to access the reward through the drinking window with an innocuous module. During the baseline measurements, the Tgp35 and WT FVBN control mice did not show any aversive behavior, and there was no difference in the number of beam breaks and time the animals spent with the licking recorded (Figure 3A). After the completion of five different training sessions, we observed the effects of the mechanical stimuli on orofacial outcome measures.

### Mechanical hyperalgesia in Tgp35 mice

In the presence of mechanical stimulators, the Tgp35 mice showed aversive behavior to mechanical stimuli as indicated by a decrease in the number of attempts to access the reward and the contact time compared to the WT FVBN mice. The Tgp35 mice exhibited significant mechanical hyperalgesia when subjected to orofacial stimulation using plates with either 6 + 6 wires (Figure 3B), 9 + 9 wires (Figure 3C), or 13 + 13 wires (Figure 3D). The Tgp35 mice made significantly fewer attempts to acquire the reward and spent much less time licking the reward compared to the wild-type controls (p < 0.001). The mechanical hyperalgesia caused by the plates with the highest number of wires produced a significantly lower reward intake as well as reduction in the licking time, compared to the plates with the lower numbers of the wires. We determined that the specific behavioral changes between the Tgp35 and the wild-type mice in the licking episodes were caused by the induction of nociception in the trigeminal area (Figure 3A-D).

### Mechanical hypoalgesia in p35 knockout mice

**Figure 4:**
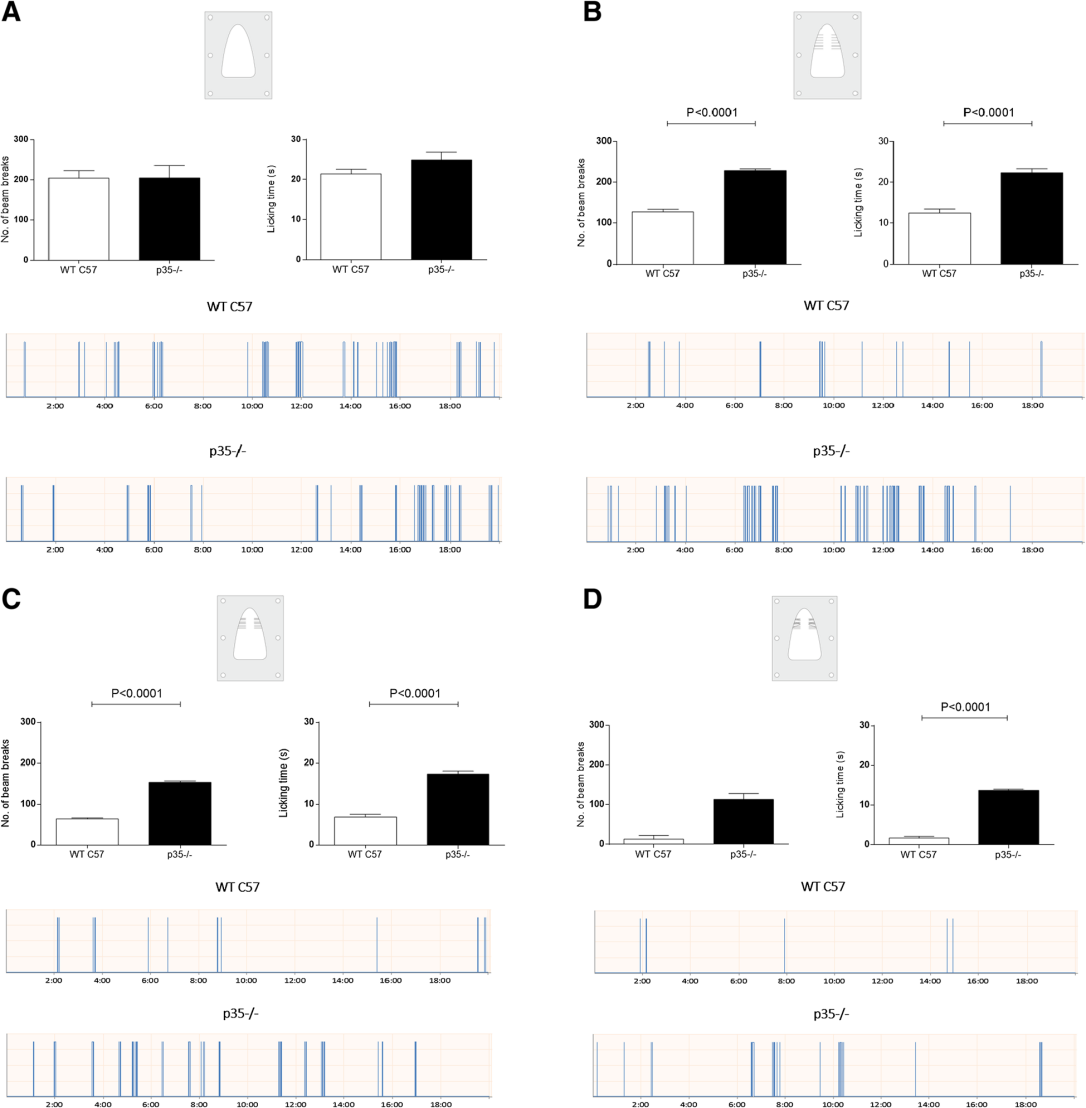
**Licking responses of the p35 knockout mice during the orofacial operant assay.** During the testing, the animals accessed the reward by inserting their snout through the plates with the different number of wires, in order to apply different force levels to the animal vibrissal pad. The number of beam breaks during the acquisition of the reward together with the licking time was measured: **(A)** without any stimulation, **(B)** using the plate with 6 + 6 wires, **(C)** 9 + 9 wires, and **(D)** 13 + 13. The data are expressed as mean ± SEM from four different animals, measured five times in case of the baseline, and three times after induction of the trigeminal nociception (unpaired *t*-test, ***p < 0.001). Two sample traces show the automatic recordings of drinking behavior in a testing period of 20 minutes in a single control (upper trace) and single p35 knockout mouse (lower trace).

Similar to the Tgp35 mice, there were no changes in the baseline reward licking paradigm in the p35−/− mice compared with the wild-type C57 control mice (Figure 4A). However, we found that the p35−/− mice displayed a significant mechanical hypoalgesia as compared to the wild-type mice when tested with the plate containing 6 + 6 wires (Figure 4B; p < 0.001). An additional noxious stimulus caused a significant decrease in the number of attempts to get the reward and time spent licking reward by the wild-type control mice compared to the p35−/− mice (Figure 4C; p < 0.001). The most obvious difference was noticed using the plate with the highest number of the wires. The wild-type mice spent only 1.7 seconds licking, whereas the p35−/− mice licked for more than 13 seconds, indicating much lower pain sensation in the p35−/− mice (Figure 4D; p < 0.001). The number of attempts and the licking time decreased significantly in the case of the wild-type mice over the entire test period. However, there were no changes in the licking patterns of the p35−/− mice after inducing the mild pain, and more obvious changes were observed inducing more painful conditions. In comparing the wild-type and the p35−/− mice, there were also clear changes in the licking pattern episodes caused by different nociceptive stimulation (Figure 4A-D).

## Discussion

The present study shows that Cdk5 has an important role in orofacial pain signaling, and that this kinase is associated with mechanical nociception in the mouse vibrissal pad. We utilized two sets of mice with significantly altered Cdk5 activity to confirm its association with orofacial pain. Both p35 knockout (with residual Cdk5 activity) and transgenic p35 (with significantly increased Cdk5 activity) mice depicted an altered response towards the mechanical stimulation. When tested with mechanical stimuli, the mice lacking the p35 gene showed hypoalgesia, whereas the mice overexpressing p35 hyperalgesia. Thus, these results clearly establish a correlation between Cdk5 activity and mechanical nociception.

Since the discovery of Cdk5, numerous studies have revealed its multifunctional roles in important physiological processes, such as brain development and function, neuronal migration, synaptic plasticity, memory, learning, and neurodegenerative disease processes [19-23,26,33]. Our previous studies demonstrated that p35, as well as Cdk5, are expressed in the dorsal root and the trigeminal ganglia, and that the expression and activity of Cdk5/p35 is increased during the inflammation. We and others have also reported that Cdk5 is required for the basal responses to noxious heat [30,31]. The p35 knockout mice showed delayed responses to the painful thermal stimulation whereas the mice overexpressing p35 were more sensitive to painful thermal stimulation at the hind paws and tail. Moreover, the inhibition of Cdk5 activity in the cultured DRG neurons attenuates the capsaicin evoked calcium influx, thus indicating a close link between Cdk5 and TRPV1 [30]. This link was further confirmed using the nociceptor-specific Cdk5 conditional knockout mice, which developed thermal hypoalgesia associated with reduced phosphorylation of TRPV1 [30]. In the current study, we extended our analysis to the role of Cdk5 in orofacial pain.

There are several animal models for studying the different types of pain; whether acute or chronic. Despite this, there is still a paucity of animal models to study orofacial pain, especially in mice. The majority of the behavioral pain tests in the orofacial area are based on pain-related spontaneous behavior. All these tests induce the pain by an injection of various chemicals (formalin, carrageenan, or capsaicin) into the upper lip or the vibrissal pad, and observe licking or grooming behavior. Recent studies use a mouse grimace scale for the measurement of short term nociception [34] or a device that quantifies a gnawing function in the mouse [35]. However, all of the current protocols for studying orofacial pain have many limitations, including variation in subjective observations, inability to escape from a noxious stimulus, and the induction of stress in the test animal. All of these can cause a large variation in the measured results.

The operant behavioral assay developed and introduced by Neubert in 2005 [16] shows that use of a reward-aversion paradigm offers more benefits. This model is based on the reward-conflict paradigm, where the test animal can decide between receiving a reward, or it can escape from the aversive stimulus by which it can control and modify its own behavior. Therefore, as compared to the other orofacial behavioral tests the use of this operant assay reduces the stress in the testing animals, there is a possibility to provide the multiple measurements using the same animal, and most importantly, it is free from investigator bias when it comes to evaluating the results that are recorded automatically. To the best of our knowledge, there is no reported study using orofacial mechanical stimulation test in mice, and we believe our present study will fill this void.

Although there are many common features in pain transduction and processing in the trigeminal and spinal systems, there are key differences in the anatomical and functional features of the primary afferent neurons of the trigeminal ganglia that distinguish them from neurons of the spinal dorsal root ganglia. Recent studies have shown that not only anatomical, but also electrophysiological and pharmacological differences [36-38] of the trigeminal afferents innervating unique target tissues such as meninges, cornea, teeth, oral/nasal mucosa, and the temporomandibular joint. These differences are consistent with our observations. It has been reported that the intrathecal administration of roscovitine, a Cdk5 inhibitor, inhibited Cdk5 activity and attenuated a formalin-induced nociceptive response in rats [39]. However, we did not observe any changes in trigeminal p35 mRNA and protein levels, nor in the Cdk5 activity after the vibrissal formalin injection (data not shown), thus supporting the theory about the differential regulation of nociception at the periphery and in the vibrissal pad.

We do not know the precise molecular mechanism by which Cdk5 activity can affect the orofacial nociception. There are several possibilities. First, the activation of the TRPV1 channel by Cdk5-mediated phosphorylation could participate in this mechanism. The TRPV1 receptor, consistent with its role as a pain regulator, is expressed in the peripheral and central nervous systems involved in pain detection, transmission, and regulation. Phosphorylation and dephosphorylation reactions regulate TRPV1 receptor activity, which is crucial in promoting inflammatory pain [40,41]. There is clear evidence that the TRPV1 channel activation at the periphery is involved in the development of inflammatory thermal hyperalgesia and heat sensitivity [42]. We have also previously reported that Cdk5 modulates thermal, nociceptive signaling through the phosphorylation of TRPV1 at threonine-407 [30]. Another recent study points out that Cdk5 can control TRPV1 membrane trafficking, and thus regulate the heat sensitivity of the nociceptors [43]. Furthermore, the systemic or intrathecal administration of TRPV1 antagonists is effective in reducing both thermal hyperalgesia, as well as mechanical allodynia associated with chronic or neuropathic pain [44-46], which indicates that TRPV1 could play an important role in integrating multiple pain-producing stimuli. More recent studies have uncovered the involvement of TRPV1 in the central mechanical nociception together in connection with the other TRP channel – TRPA1 [47]. Other studies speculate that the central mechanical hyperalgesia could be induced by the functional interaction between P2X3 [48] or NMDA receptor [49] and TRPV1. These studies provide the evidence that TRPV1 channels are important not only for the peripheral pain sensation, but they can also play an important role in the central mechanical nociception.

An interesting possibility is that Cdk5 can mediate orofacial mechanical hyperalgesia through the regulation of the neurotransmitter release, thus indicating that this kinase could be an important presynaptic control parameter. Deregulation of its activity could affect nociception via the presynaptic mechanism with the subsequent initiation of the pain sensation [50-52]. Another possibility is that Cdk5 could mediate the orofacial mechanical hyperalgesia through the activation of other potential mechanotransducers. It is well known that the upregulation of Cdk5 activity can lead to phosphorylation of delta opioid receptor [53,54], NMDA receptor [55,56], P2X3 receptor [57], and voltage gated calcium channels [58]. Additionally, there are other potential candidates like TRPA1 [59-61] or TREK channels [62,63] that contain the Cdk5 phosphorylation consensus sequence and may be involved in the Cdk5-mediated activation and mechanotransduction in the orofacial area. To understand the precise mechanism through which Cdk5 regulates orofacial mechanosensitisation will require further studies; including molecular, electrophysiological, and behavioral methods to map the functional role of Cdk5 in this type of the nociception.

## Conclusions

We have adapted orofacial stimulation test for mice that could be used for orofacial pain studies, and using this test we have identified that Cdk5 activity has an important role in orofacial mechanical nociception. Moreover, our studies also demonstrate that genetically engineered mice with the altered Cdk5/p35 levels will prove to be valuable models to identify and characterize the inhibitors of Cdk5/p35 as novel analgesics to treat orofacial pain.

## Methods

### Animals

The p35−/− mice and the age-matched, wild-type controls were maintained in C57BL6/129SVJ background. Tgp35 mice and the wild-type littermate controls were maintained in FVBN background. All of the animals were housed and bred in standard cages, and they were maintained in climate and light controlled rooms with free access to food and water in accordance with the U.S. National Institutes of Health Guide for Care and Use of Laboratory Animals. All of the experiments adhered to the guidelines of the IASP Committee for Research and Ethical Issue [64].

### Rotarod test

The p35−/− and Tgp35 mice were evaluated for motor abilities, coordination, and balance by performance on the rotarod (Model 7650, Ugo Basile, Italy). This instrument consists of a platform under a rotating cylinder that can be set either to accelerate from 4 to 40 rpm in the defined period of time or used at a constant speed. The automated rotarod system has a timer linked to the platform panel onto which the mouse falls (approximately 15 cm below the rotating cylinder). During the testing, the animals have to continuously walk forward to keep from falling off the rotating cylinder. After the mice became familiarized with this procedure, they were placed on the rotating cylinder, and the time until the animal fell down was measured in three different tests performed on three consecutive days.

### Open field test

Anxiety, exploratory activity, as well as spontaneous motor activity were evaluated using the VersaMax Animal activity monitoring system (AccuScan Instruments Inc., Columbus, OH, USA). The testing instrument consists of a clear, plexiglass, rectangular cage (42 × 42 × 30 cm), with the transparent side wall. Two sets of sixteen photocells are aligned from the front to back of the cage and from side to side for recording horizontal activity. Vertical activity is assessed by an additional sixteen photocells located above the horizontal cells. The mice were placed individually in the center of the open field apparatus, and their behavior was monitored for ten minutes. Horizontal or vertical activity (rearing measured by counting the number of beam interruptions), time spent in the center area of the open field, and stereotypic counts were automatically recorded using the VersaMax software system.

### Mouse orofacial stimulation test

Using a small plastic reducer, we have modified the Orofacial Stimulation Test (31300, Ugo Basile, Italy), which was previously used only for the measurement of hypersensitivity to thermal or mechanical stimulation of the trigeminal area in rats, and adapted it for assessing behavioral responses to mechanical stimulation in mice (Additional file 1C).

An orofacial stimulation test consists of several parts - the plastic cage (26.5 cm wide, 20 cm deep, and 48 cm long) with the interface wall containing a drinking window (1.5 cm wide × 2 cm high) that allows a mouse an access to a reward; an infrared photo beam built on the exterior aspect of the window linked to the ORO software that automatically quantifies feeding behavior by measuring the number of attempts the animals made to acquire the reward, and the total duration of feeding time (Additional file 1A). The equipment is supplemented with metal inserts containing a different number and configuration of the Nitinol wires (0,155 mm) utilized to induce varying degrees of trigeminal, mechanical nociception (Additional file 1B). Each of these metal inserts can be mounted onto the opposite aspect of the drinking window with six screws.

During the orofacial operant assay, the test mouse inserts its snout through the drinking window in the interface wall to access a nozzle of the bottle containing reward (30% sucrose) [65]. Simultaneously, while accessing the nozzle, the mouse breaks an analog output from the infrared detectors (Banner Engineering, Minneapolis, MN, USA) placed directly behind the drinking window, and thus its attempts to get the reward can be recorded and analyzed with the ORO software (Additional file 1D).

The mice were deprived of food and water for at least a period of eight hours prior to the testing sessions, which was done to increase the incentive for reward acquisition. After the completion of tests, access for food and water was restored. The mice were trained and tested in the same cage and at the same time each day, and they had a day of rest between the testing.

Initially, the animals were trained to access the reward through the blank plate (without the wires). For mechanical testing, access to the reward was impeded by inserting the plate with the different number of the wires providing mechanical contact with the vibrissal pad region of the tested animal (Additional file 1D). We used three different plates with 6 + 6, 9 + 9, or 13 + 13 wires at each side in order to apply a different level of mechanical force to the animal vibrissal pad, so as to induce different degrees of pain. After completing five, different, twenty-minute training sessions, the animals were retested three times using each plate with a different number of wires, in increasing order, starting with the lowest to the highest number of wires. The outcome measures that were collected during the testing consisted of the total duration of time the test mouse spent acquiring the reward over a twenty minute period of time, with or without (baseline measurements) mechanical stimulators and the number of attempts the test mouse made to access the reward. Both were determined automatically by an interruption of an infrared beam when the animal placed its snout through the opening in the interface wall.

### Quantitative real-time PCR (q-PCR)

The trigeminal ganglia, brainstem, and the brain were dissected out from p35−/−, Tgp35, and wild-type control mice and these tissues were immediately frozen at −80°C. The total RNA was extracted using the RNeasy Mini Kit (Qiagen, Valencia, CA, and USA), according to the manufacturer’s instructions. The purity of the RNA was assessed by the ratio of absorbance at 260 nm and 280 nm. The RNA from each sample was reverse transcribed using a High Capacity cDNA Reverse Transcription Kit (Applied Biosystems, Foster City, CA, USA).

The q-PCR reactions were conducted using cDNA, specific primers (Assays on Demand Gene Expression Products), and TaqMan Universal PCR Master Mix (Applied Biosystems, Foster City, CA, USA), and they were run in duplicates using the Real-time PCR System 7500 (Applied Biosystems, Foster City, CA, USA). The p35 mRNA levels were normalized to the levels of HPRT using the comparative cycle threshold (ct) method.

### Antibodies

Anti-p35 (C19), Anti-Cdk5 (C8) and secondary horseradish peroxidase-conjugated anti-mouse and anti-rabbit antibodies were obtained from Santa Cruz Biotechnology (Santa Cruz Biotechnology, Santa Cruz, CA, USA). The anti α-tubulin antibody was purchased from Sigma Aldrich (Sigma-Aldrich, St. Louis, MO, USA).

### Western blotting

The tissue homogenates were lysed in tissue protein extraction reagent (Thermo Scientific, Rockford, IL, USA) containing a cocktail of protease (Complete Mini, Roche, Indianapolis, IN, USA) and phosphatase (PhosSTOP, Roche, Indianapolis, IN, USA) inhibitors to avoid degradation of the proteins. After thirty minutes of incubation on ice, the samples were spun down at 14000 rpm at 4°C for 30 min. The supernatant was assayed for total protein concentration using the Bradford Protein Assay (Bio-Rad, Hercules, CA, USA). The proteins were denatured by boiling them with NuPAGE LDS sample buffer and NuPAGE sample reducing agent (Invitrogen, Carlsbad, CA, USA) for 10 min. Each sample was separated by 4-12% SDS PAGE gels (Invitrogen, Carlsbad, CA, USA) and transferred to 0.45 μm nitrocellulose membrane (Invitrogen, Carlsbad, CA, USA). The blots were blocked for 1 h in phosphate buffered saline containing 5% nonfat dry milk and 0.05% Tween20 (Sigma-Aldrich, St. Louis, MO, USA), and then they were blotted with primary antibodies overnight at 4°C. The membranes were then probed with horseradish peroxidase-conjugated anti-mouse or anti-rabbit IgG at room temperature for one hour, and they were finally developed by SuperSignal West Pico or Dura Chemiluminescent Substrate (Thermo Scientific, Rockford, IL, USA). The immunoblots were analyzed by densitometry using ImageJ analysis system software.

### Immuno-precipitation and Cdk5 activity assay

Immuno-precipitation and Cdk5 kinase activity were performed as described previously [66]. Briefly, the protein G (+) A-agarose beads were washed three times with tris-buffered saline (TBS) and incubated with Cdk5 antibody (1–2 ug/500 ug of protein lysate, Santa Cruz Biotechnology, Santa Cruz, CA, USA) for 1 h at room temperature with gentle mixing. The beads were centrifuged and washed three times with TBS and then suspended in TBS. The protein lysates from the trigeminal ganglia, brainstem, and brain were incubated with antibody conjugated beads for 2 h and 30 min at 4°C on a rotating wheel. The beads were subsequently centrifuged and washed two times with TBS, one time with 1X kinase buffer and suspended in kinase buffer (50 mM Tris/HCl pH 7.4, containing 1 mM EGTA, and 5 mM MgCl2). The immunoprecipitated beads were used as an enzyme source for the kinase activity. For the kinase assay, a total volume of 50 μL of kinase assay mixture was used, containing 50 μM Tris/HCl (pH 7.4) with EGTA, 1 mM dithiothreitol, 5 mM MgCI2, 10 μg of histone H1, and 10 μL of Cdk5 immunoprecipitates. The phosphorylation reaction was initiated by the addition of 0.1 mM [γ-^32^P] ATP and incubated at 30°C for one hour. The reaction was stopped by the addition of the Laemmli sample buffer. The reaction mixture was heated for five minutes at 90°C and electrophoresed on a 4-20% SDS-PAGE gel stained with Coomassie blue, and then dried and exposed overnight for the detection of ^32^P-labeled Histone H1 by autoradiography. The films were scanned, and the bands were quantified using ImageJ software.

### Statistical analysis

All data are expressed as a mean ± SEM. The statistical evaluation was done with GraphPad Prism software, version 5.0 (GraphPad, San Diego, CA, USA). Statistical differences between the experiments were assessed by unpaired *t*-test. The significance level was set at p < 0.05.

## Abbreviations

Cdk5: Cyclin-dependent kinase 5; TRPV1: Transient receptor potential vanilloid 1; TG: Trigeminal ganglia; Tgp35: Transgenic p35 mice; p35-/-: p35 knockout mice; q-PCR: Quantitative real-time PCR; WT: Wild-type.

## Competing interest

The authors declare that they have no conflict of interest.

## Authors’ contributions

MP and ABK designed the experiments. MP, AT, EU, BH, and NDA performed research; MP, AT, EU, BH, NDA, HCP and ABK analyzed data, and MP and ABK wrote the manuscript. All authors read and approved the final manuscript.

## Supplementary Material

Additional file 1 **Mouse orofacial stimulation test system used for the measurement of orofacial mechanical nociception. (A)** Orofacial stimulation test device. **(B)** The mechanical inserts with different number of wires used for the induction of trigeminal pain. **(C)** The plastic reducer used for the modification of the existing system to be applicable for the characterization of mouse orofacial pain. **(D)** An example showing the mouse during the reward licking while its vibrissal region is in direct contact with the wires. Click here for file

Additional file 2 **Effect of different p35 genotype on locomotor and exploratory activity. (A)** The mean performance time determined as time spent on the rotating cylinder during the acceleration. **(B)** The latency to fall from the rotating cylinder by the constant speed. **(C)** The unaffected horizontal and **(D)** vertical activity as revealed by the open field test. The data analyzed by the unpaired t-test are expressed as mean ± SEM and represent the mean from four different animals. Click here for file

Additional file 3 **The effect of upregulated p35 and Cdk5 activity on mouse behavior in an open-field test. (A)** The center distance travelled and time spent in the center of the activity cage, **(B)** stereotypy and the time Tgp35 mice spent with the stereotypic behavior. **(C)** The time Tgp35 mice spent in the different parts of the activity cage during ten minutes of measurement. These values represent the mean ± SEM from four animals. Click here for file

Additional file 4 **The effect of downregulated p35 and Cdk5 activity on mouse behavior in an open-field test. (A)** The center distance travelled and time spent in the center of the activity cage, **(B)** stereotypy and the time p35 knockout mice spent with the stereotypic behavior. **(C)** The time p35-/- mice spent in the different parts of the activity cage during 10 min of measurement. These values represent the mean ± SEM from four animals. Click here for file
