# Supplementary figures and images for: Activation of cyclin-dependent kinase 5 mediates orofacial mechanical hyperalgesia
Source: Mol Pain. 2013 Dec 21;9:66. doi: 10.1186/1744-8069-9-66 (PMC3882292; doi:10.1186/1744-8069-9-66)

Additional Figure 1

A

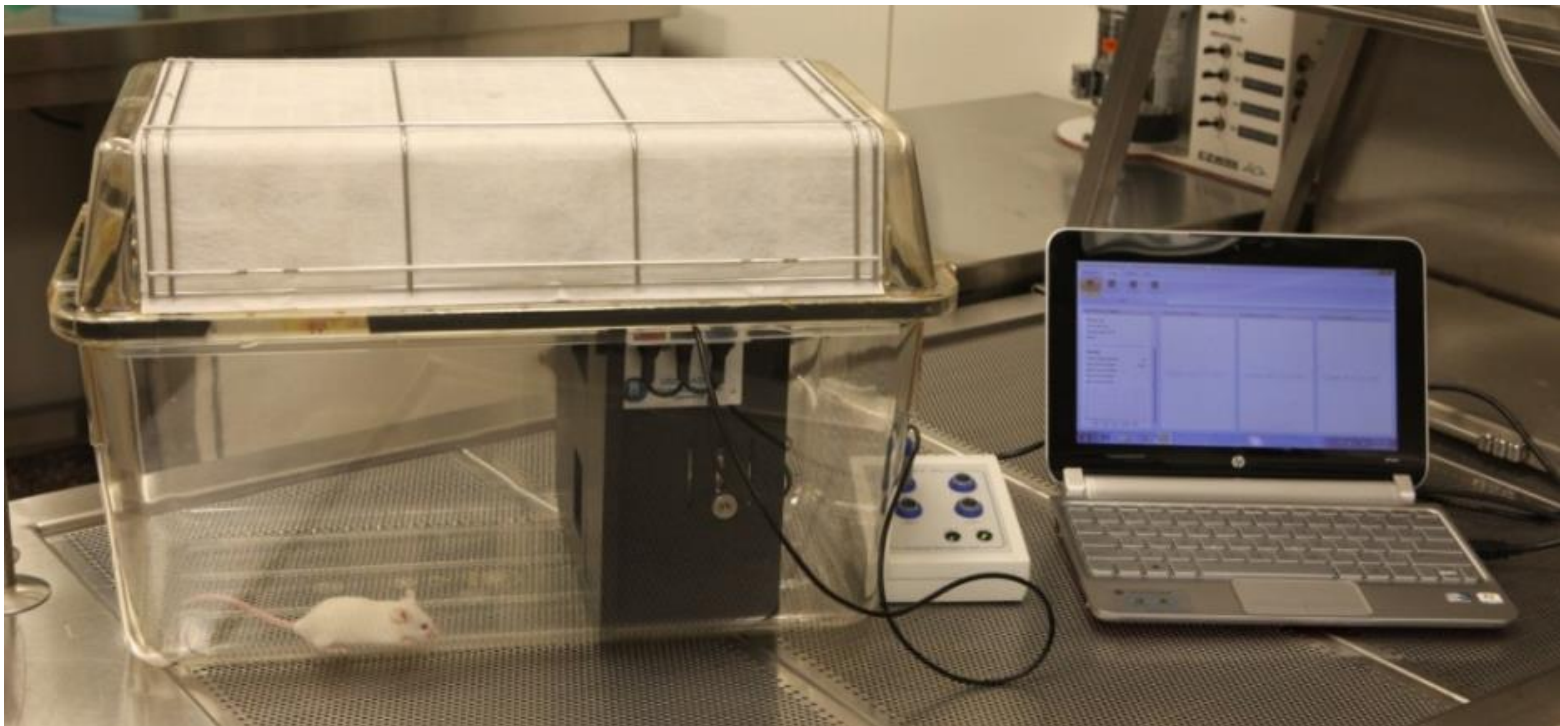

B

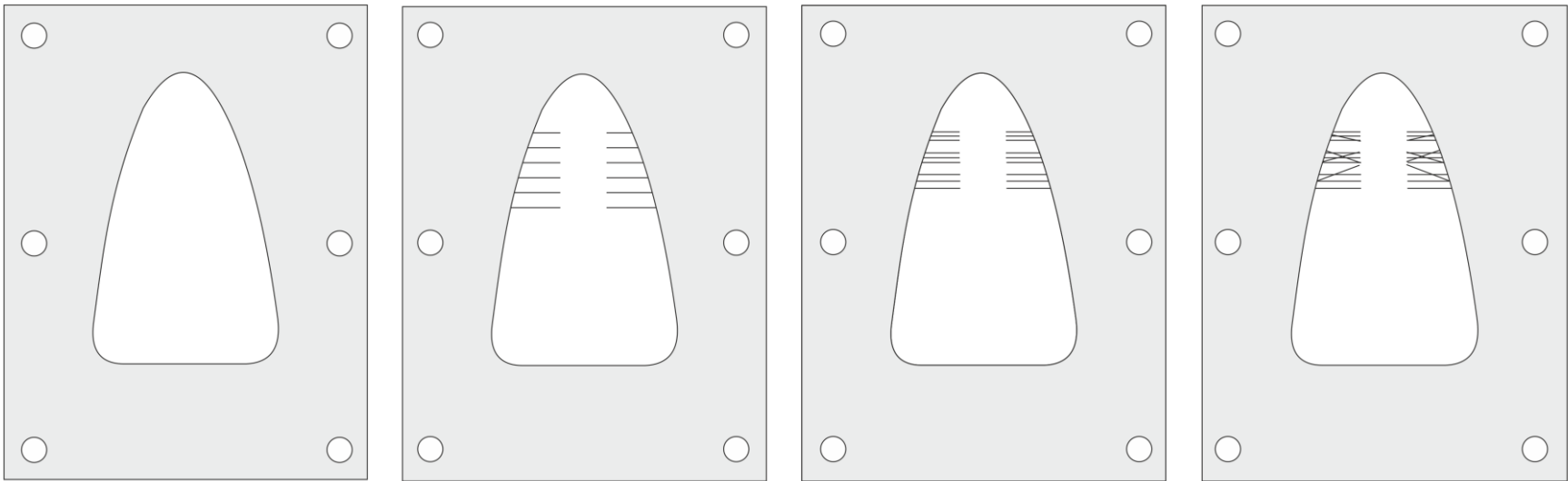

C

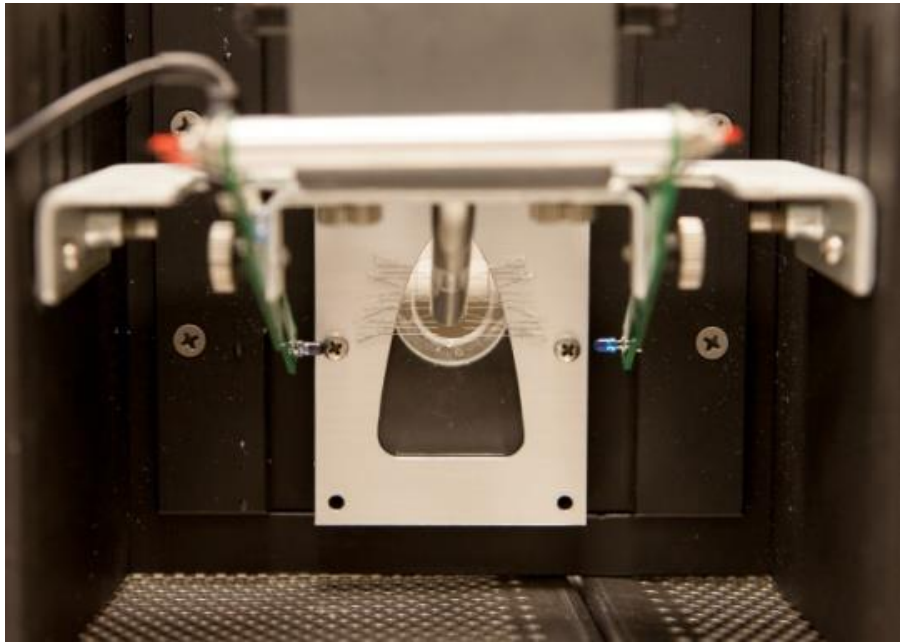

D

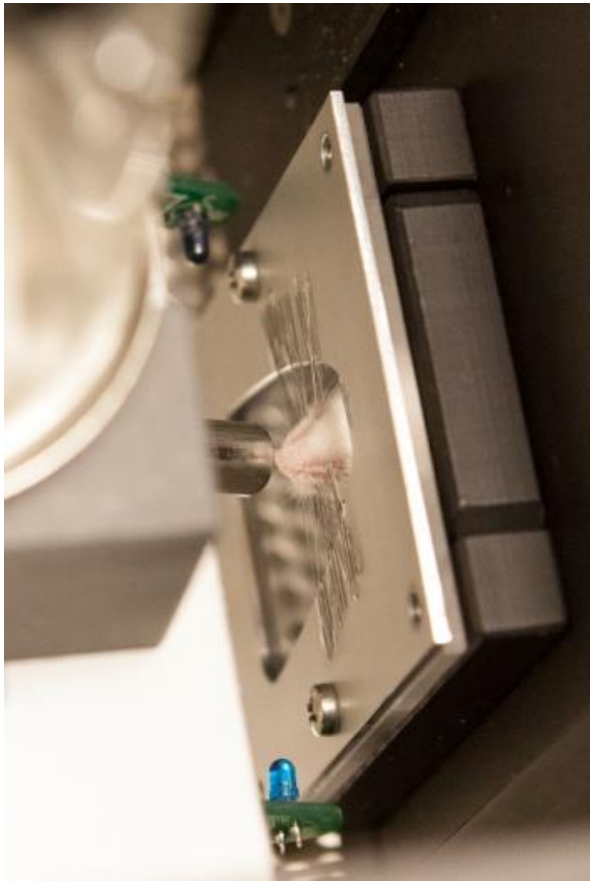

Supplement: Additional file 1 — Mouse orofacial stimulation test system used for the measurement of orofacial mechanical nociception. (A) Orofacial stimulation test device. (B) The mechanical inserts with different number of wires used for the induction of trigeminal pain. (C) The plastic reducer used for the modification of the existing system to be applicable for the characterization of mouse orofacial pain. (D) An example showing the mouse during the reward licking while its vibrissal region is in direct contact with the wires. [file 1744-8069-9-66-S1.pdf]

Additional Figure 2

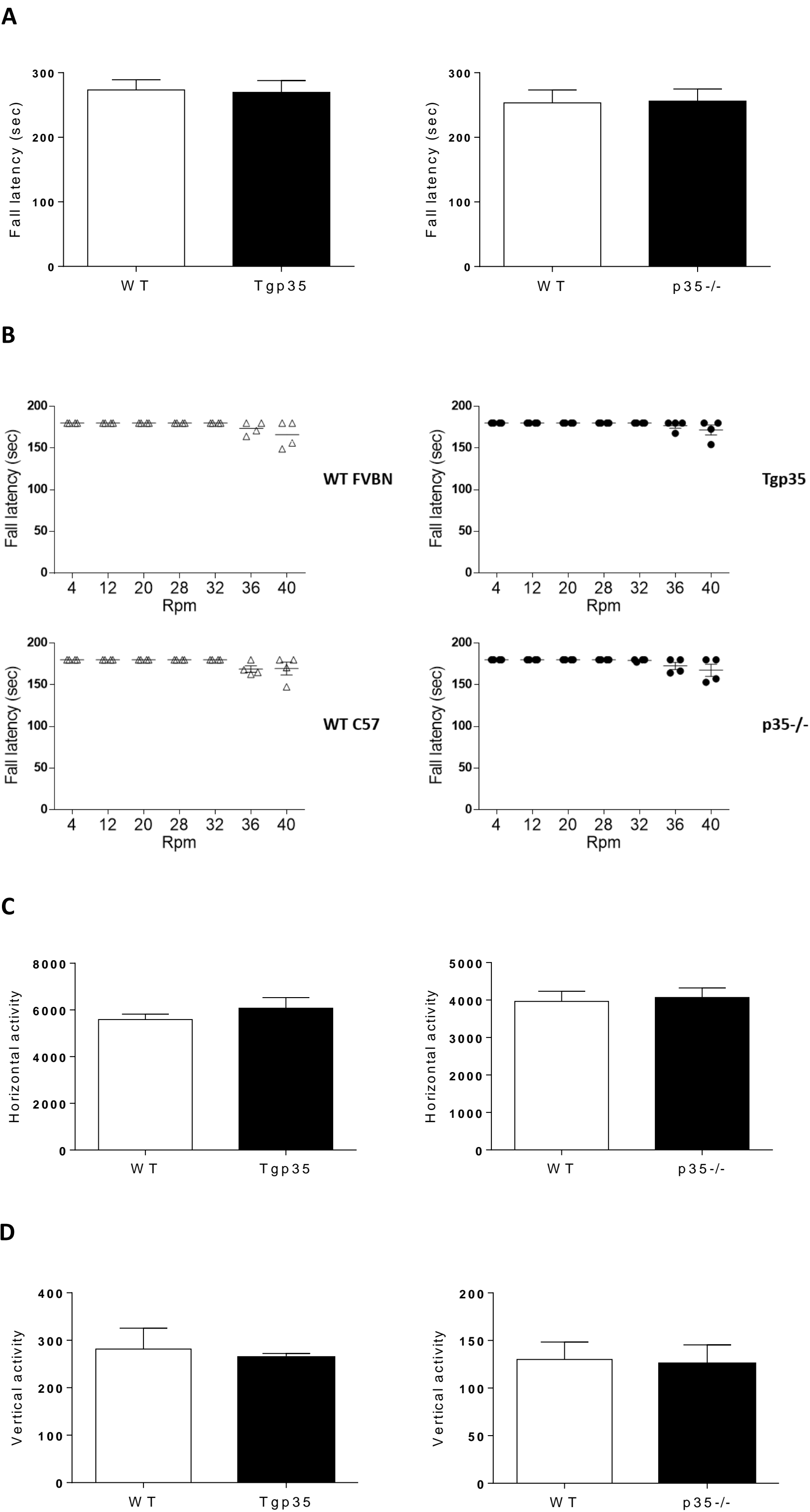

Supplement: Additional file 2 — Effect of different p35 genotype on locomotor and exploratory activity. (A) The mean performance time determined as time spent on the rotating cylinder during the acceleration. (B) The latency to fall from the rotating cylinder by the constant speed. (C) The unaffected horizontal and (D) vertical activity as revealed by the open field test. The data analyzed by the unpaired t-test are expressed as mean ± SEM and represent the mean from four different animals. [file 1744-8069-9-66-S2.pdf]

Additional Figure 3

A

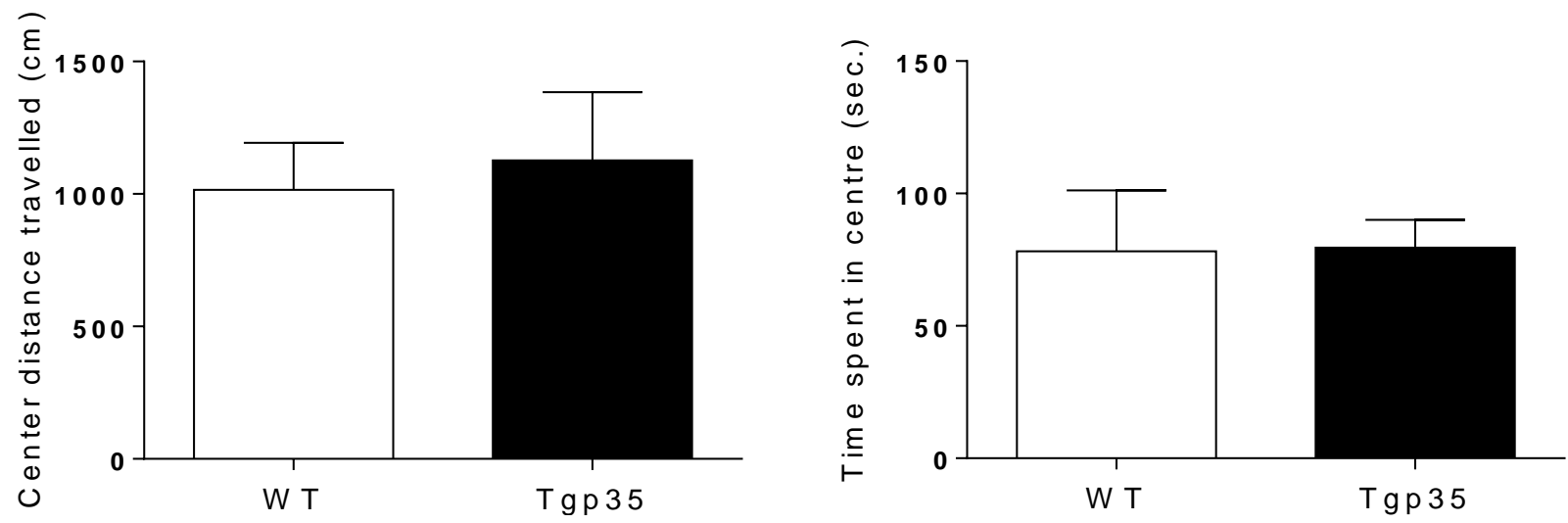

B

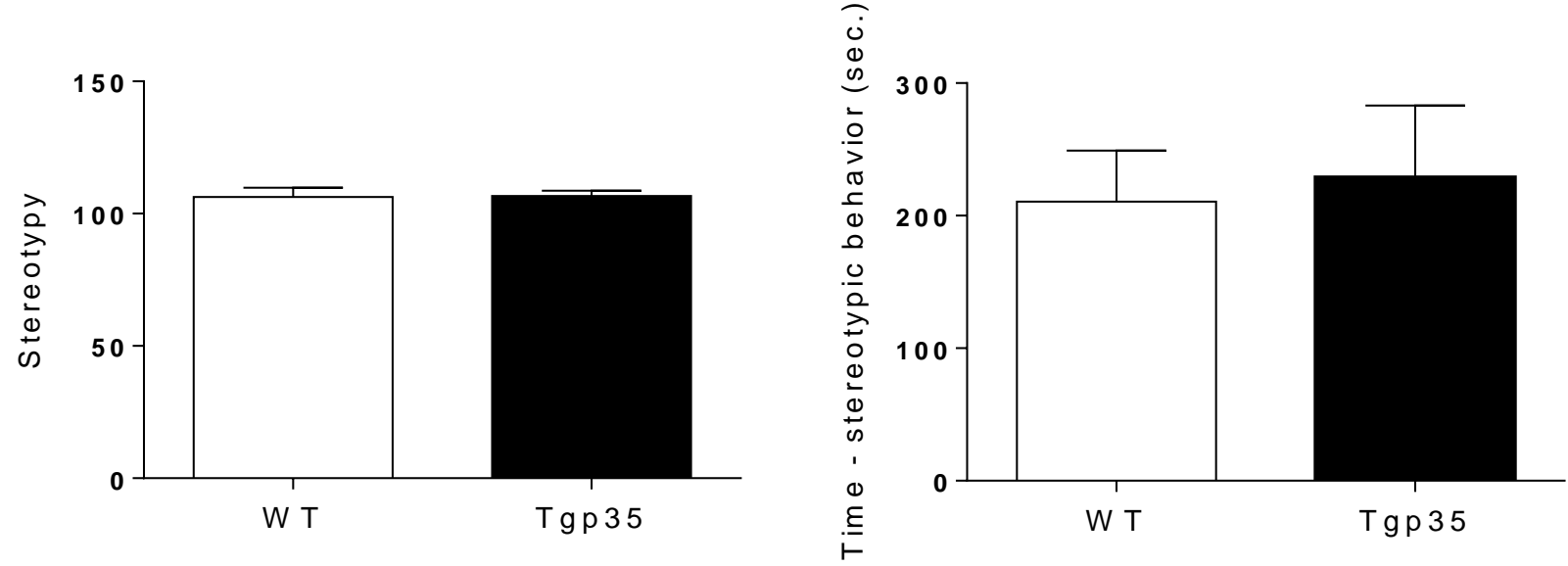

C

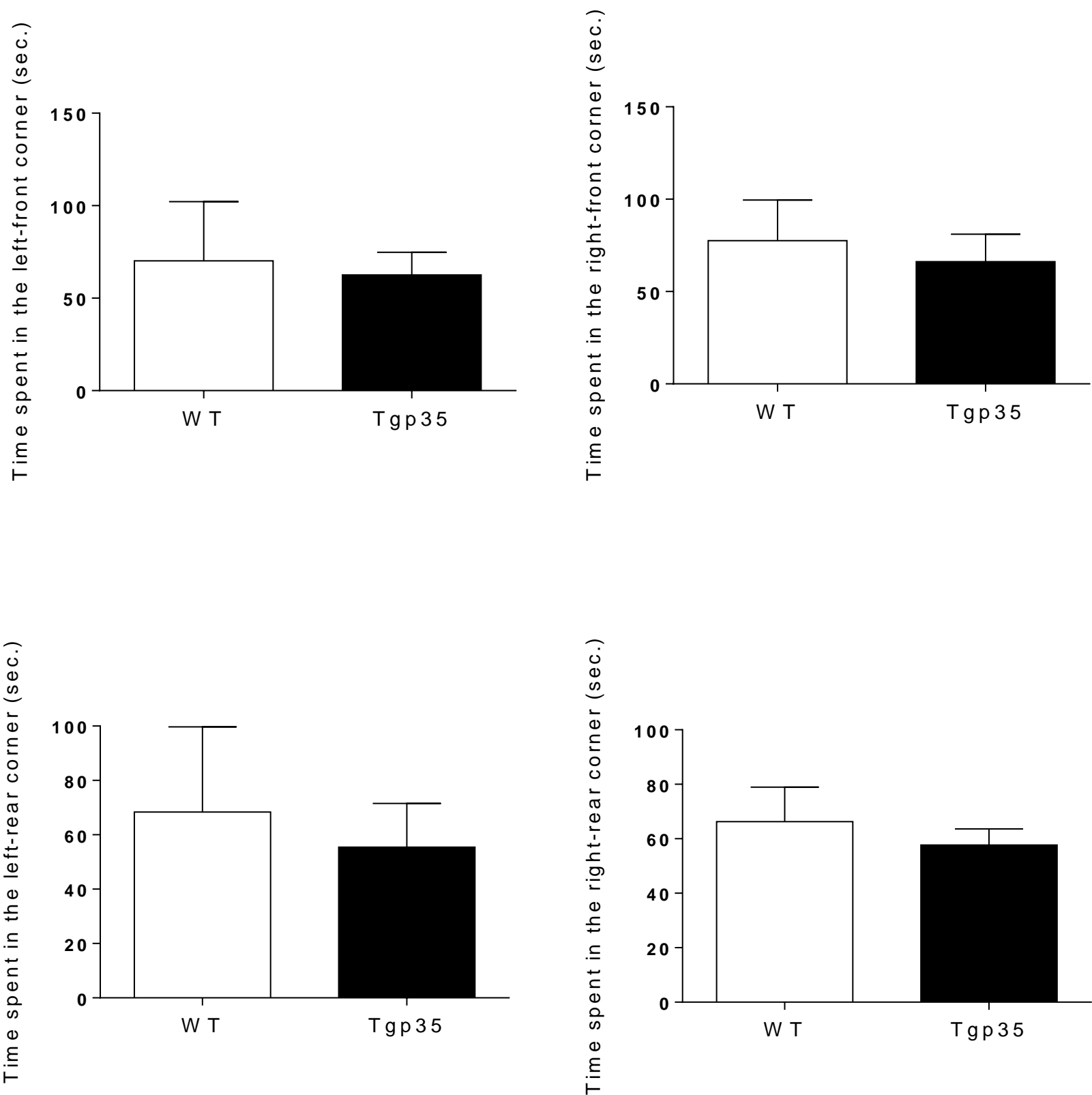

Supplement: Additional file 3 — The effect of upregulated p35 and Cdk5 activity on mouse behavior in an open-field test. (A) The center distance travelled and time spent in the center of the activity cage, (B) stereotypy and the time Tgp35 mice spent with the stereotypic behavior. (C) The time Tgp35 mice spent in the different parts of the activity cage during ten minutes of measurement. These values represent the mean ± SEM from four animals. [file 1744-8069-9-66-S3.pdf]

Additional Figure 4

A

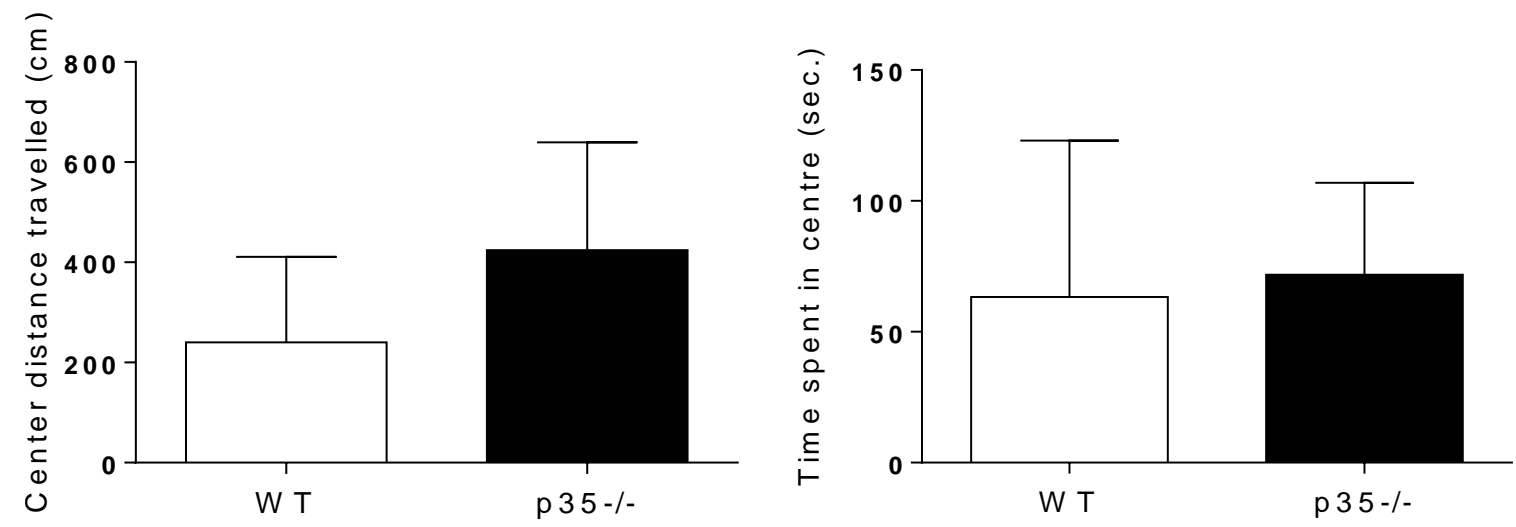

B

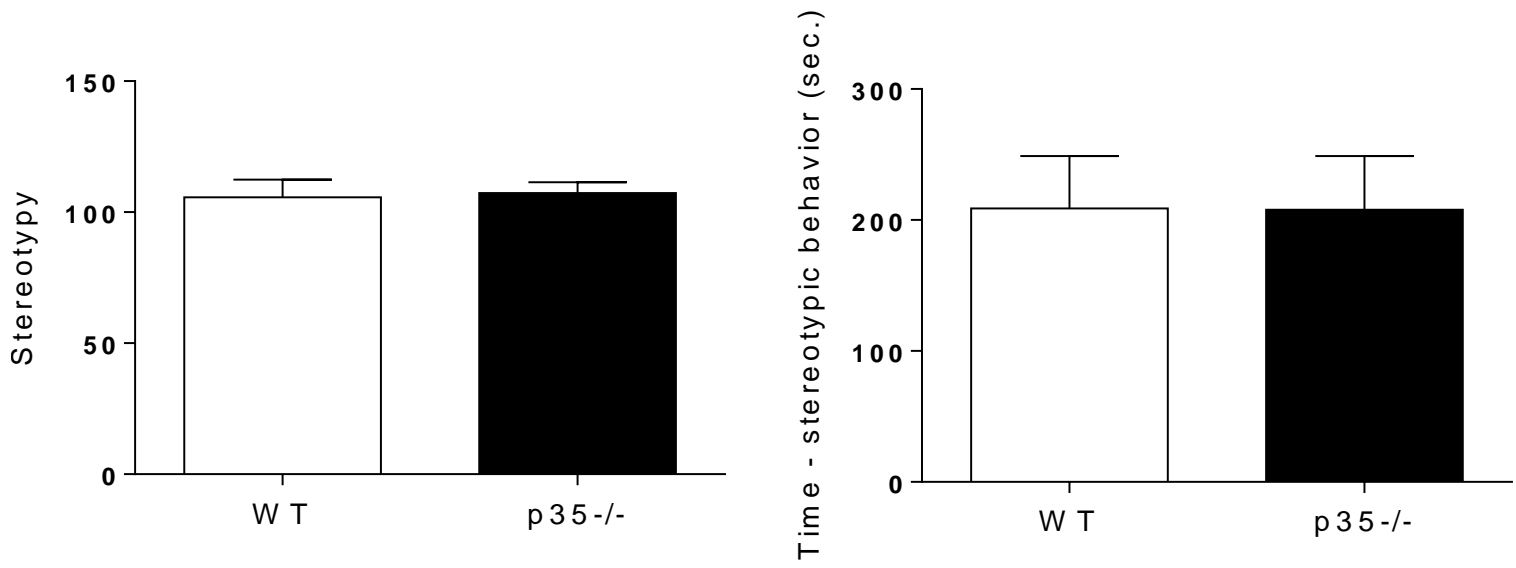

C

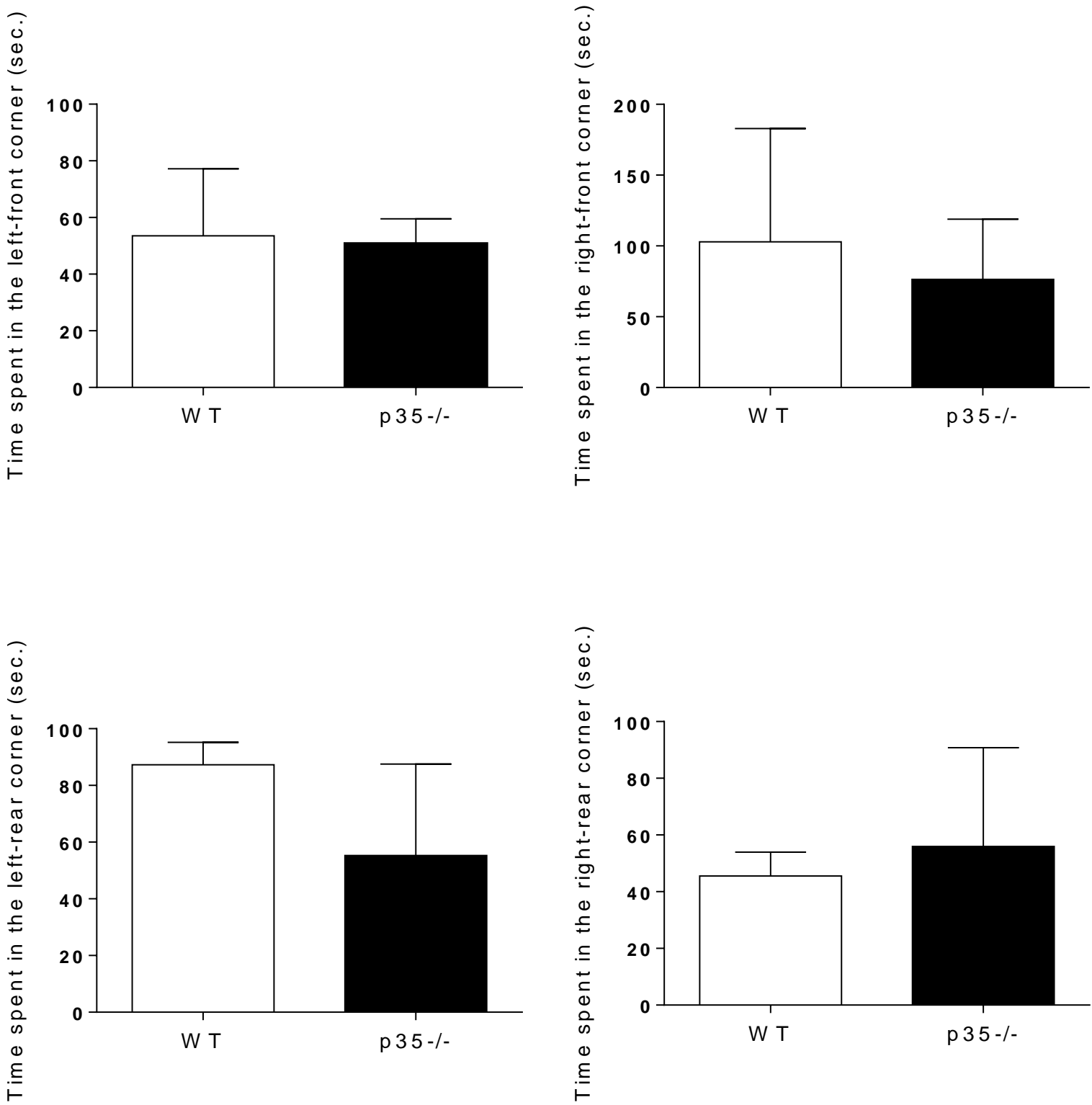

Supplement: Additional file 4 — The effect of downregulated p35 and Cdk5 activity on mouse behavior in an open-field test. (A) The center distance travelled and time spent in the center of the activity cage, (B) stereotypy and the time p35 knockout mice spent with the stereotypic behavior. (C) The time p35-/- mice spent in the different parts of the activity cage during 10 min of measurement. These values represent the mean ± SEM from four animals. [file 1744-8069-9-66-S4.pdf]
